# Graphene Oxide-Enriched Polymer: Impact on Dental Pulp Cell Viability and Differentiation

**DOI:** 10.3390/polym17131768

**Published:** 2025-06-26

**Authors:** Magdalena Vega-Quiroz, Agustin Reyes-Maciel, Christian Andrea Lopez-Ayuso, Carlos A. Jurado, Hector Guzman-Juarez, Carlos Andres Alvarez-Gayosso, Benjamin Aranda-Herrera, Abdulrahman Alshabib, Rene Garcia-Contreras

**Affiliations:** 1 Interdisciplinary Research Laboratory, Nanostructures, and Biomaterials Area, Escuela Nacional de Estudios Superiores (ENES) Unidad León, Universidad Nacional Autónoma de México (UNAM), León 37684, Guanajuato, Mexico; 2 Division of Operative Dentistry, Department of General Dentistry, College of Dentistry, The University of Tennessee Health Science Center, Memphis, TN 38103, USA; 3 School of Dental Medicine, Ponce Health Sciences University, Ponce, PR 00732, USA; 4 Osforma, Los Algodones 23895, Baja California, Mexico; 5 Department of Restorative Dentistry, College of Dentistry, King Saud University, Riyadh 11545, Saudi Arabia

**Keywords:** graphene oxide, cytotoxicity, cell proliferation, polymethylmethacrylate

## Abstract

Background: Reconstructing maxillofacial defects is important in dentistry, so efforts are being made to develop materials that promote cell migration and repair. Graphene oxide (GO) is used to enhance the biocompatibility of polymethylmethacrylate (PMMA) due to its nanostructure. Objective: to assess cytotoxicity, cell proliferation, and differentiation of human dental pulp stem cells (hDPSC) in response to a conventional PMMA (PMMA) and polymer enriched with GO (PMMA+GO). Methods: Experiments were carried out with primary hDPSC subcultures. The PMMA and PMMA+GO were tested in direct and indirect contact. Cytotoxicity (1 day) and proliferation (3, 7, and 14 days) were evaluated with an MTT bioassay. The osteogenic, adipogenic, and chondrogenic aspects were determinate with alizarin red, oil red, and safranine. Mean values, standard deviation, and percentages were calculated; data were analyzed with Shapiro–Wilks normality and Student’s *t*-test. Results: The cell viability of PMMA and PMMA+GO in direct contact correspond to 90.8 ± 6.2, 149.6 ± 14.5 (1 day); 99.9 ± 7.0, 95.7 ± 6.1 (3 days); 120.2 ± 14.6, 172.9 ± 16.2 (7 days); and 102.9 ± 17.3, 95.4 ± 22.8 (14 days). For indirect contact, 77.2 ± 8.4, 99 ± 21.4 (1 day); 64.8 ± 21.6, 67.0 ± 9.6 (3 days); 91.4 ± 16.5, 142 ± 18.7 (7 days); and 63 ± 15.8, 79.1 ± 3.1 (14 days). PMMA+GO samples showed enhanced adipogenic, chondrogenic, and osteogenic aspects. Conclusions: The integration of GO into PMMA biopolymers stimulates cell proliferation and differentiation, holding great promise for future applications in the field of biomedicine.

## 1. Introduction

Tissue engineering is a multidisciplinary field that integrates knowledge of cell biology, biochemistry, and materials science to create biological structures capable of restoring, improving, or preserving the function of damaged tissues [1,2,3]. Thanks to advances in these areas, it has been possible to develop increasingly complex and functional biological systems [4]. This discipline is based on the coordinated interaction of three key elements [5]: The first of these is stem cells, with their unique ability to divide and differentiate into different specialized cell types [6]. The second component is the three-dimensional scaffolds, which act as support structures and create a suitable microenvironment for cell adhesion, nutrition, and proliferation [3,7]. Finally, the third element is constituted by growth factors, a group of proteins secreted by the organism itself, which play an essential role in the regulation of key biological processes, such as cell migration and differentiation, through chemical signals [5,8].

Graphene oxide (GO), a derivative of graphene obtained through the oxidation of graphite, is a material that has attracted a great deal of interest within the scientific community due to its outstanding physicochemical and biological properties [9,10]. GO belongs to the family of carbon-derived nanomaterials, a group that includes various allotropic forms. The range of forms encompasses three-dimensional structures, including diamond and graphite; two-dimensional structures, such as graphene; one-dimensional structures, such as carbon nanotubes; and zero-dimensional structures, such as fullerenes [11,12]. There are different methods to synthesize GO, among which mechanical exfoliation, chemical vapor deposition, and the Hummers method stand out. The latter is one of the most widely used and consists of chemical oxidation of graphite using sulfuric acid and potassium permanganate [13].

In terms of properties, GO is rigid, lightweight, and almost transparent, as well as showing remarkable mechanical strength. It also has higher electrical and thermal conductivity than many other materials such as dental polymers, composite resins typically used in oral applications, and ceramic-based biomaterials [14,15,16,17]. A particularly attractive feature is its ability to interact with other substances, allowing its properties to be modified according to the specific needs of different biomedical applications [15].

Since its introduction by Geim and Novoselov in 2007, its biocompatibility has been extensively studied, as well as its remarkable ability to interact with cells, facilitating key processes such as cell adhesion, proliferation, and differentiation [9,10,18].

In this particular context, GO has been identified as a potentially valuable nanomaterial, owing to its hexagonal crystalline structure, which comprises carbon atoms in a specific arrangement [1]. Due to these properties, GO has established itself as a promising biomaterial for various applications, especially in the field of tissue engineering and regenerative dentistry [19,20].

Polymethylmethacrylate (PMMA) was first introduced in 1936 as a material for the manufacture of dental prostheses. PMMA (IUPAC name: poly[1-(methoxy carbonyl)-1-methyl ethylene]) is a synthetic polymer produced through the free radical polymerization of methyl methacrylate (C_5_O_2_H_8_), resulting in poly(methyl methacrylate), a long-chain polymer represented chemically as (C_5_O_2_H_8_)ₙ. The polymerization process begins with the initiation step, where free radicals are generated using chemical initiators or external energy sources such as heat, light, or microwaves. During propagation, the reaction progresses as monomer units sequentially add to the growing polymer chain. This continues until the termination phase, where the reactive free electrons are transferred to the end of the chain, ending further growth. It is still one of the most widely used methods today, primarily due to its low cost, ease of handling, and good aesthetic performance [21,22]. However, its mechanical and biological properties present some limitations, which has prompted its modification through the incorporation of nanoparticles, such as GO. This combination not only improves the mechanical characteristics of PMMA, but also supports key cellular processes, such as the proliferation and differentiation of human dental stem cells (hDPSCs), which broadens its potential for applications in bone and tissue regeneration [23].

In this context, the aim of this research is to evaluate the effect of a biopolymer enriched with graphene oxide on the viability and differentiation of hDPSC. It is proposed that this innovative biomaterial could have applications in immediate prostheses, fixed prostheses, orthopedic devices, nasal obturators, and bone cements, offering a biocompatible and effective alternative to optimize results in regenerative dental treatments.

## 2. Materials and Methods

### 2.1. PMMA and PMMA+GO Sample Preparation

Samples of PMMA (control group) and PMMA+GO (experimental group, commercial product: Osforma Addir Pink, Los Algodones, Baja California, Mexico) were prepared according to the manufacturer’s instructions, using a 3:1 powder-to-liquid volume ratio. In the case of PMMA+GO, the GO was pre-dispersed in the polymer powder provided by the manufacturer.

For both groups, the corresponding amount of polymer was weighed, and the monomer was measured and poured into a container. The components were manually mixed with a spatula to ensure adequate wetting and minimize air incorporation. Once the mixture reached the plastic phase, it was placed into stainless-steel circular molds (1.5 mm diameter × 1 mm thickness), pressed, and left undisturbed for 30 min to complete the polymerization process.

**Figure 1:**
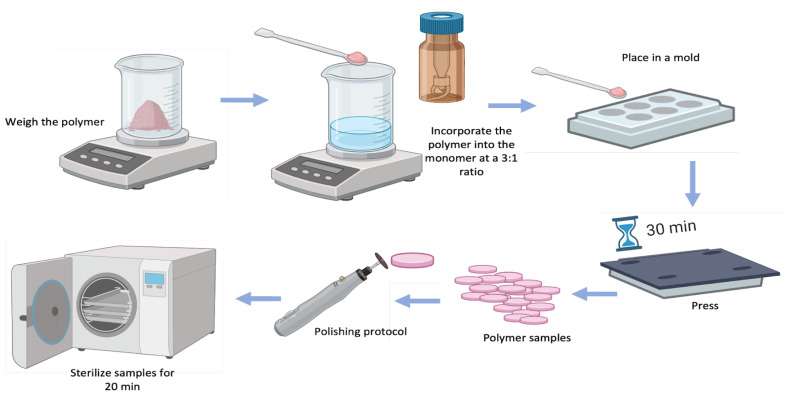
Processing of conventional and GO polymer samples. Source: Direct obtained with BioRender 2025.

Afterward, the specimens were removed from the molds, polished, and sterilized for 20 min. The samples were then stored for subsequent analysis (Figure 1).

#### 2.1.1. PMMA and PMMA+GO Characterization

The PMMA+GO group was characterized using optical microscopy and GO alone with scanning electron microscopy (SEM) (Hitachi High-Tech Corporation, Tokyo, Japan) to evaluate the morphology, distribution of graphene oxide within the polymer matrix and GO morphological characteristics. Optical microscopy was used for initial surface observations at standard magnifications (50–200×) to identify general dispersion patterns and surface texture. For GO detailed analysis, SEM was performed using a Hitachi S5500 microscope (Hitachi High-Tech Corporation, Tokyo, Japan); samples were mounted on carbon tape and examined at 1500× magnification, with an acceleration voltage of 20–25 kV and a chamber pressure of 20–25 Pa, following previously described protocols [14].

### 2.2. Cell-Culture Assays

hDPSC cells were sourced from the cell bank of the Interdisciplinary Research Laboratory (LII), part of the Nanostructures and Biomaterials Division at the National School of Higher Studies, León Campus, National Autonomous University of Mexico (UNAM), in Guanajuato, Mexico. The study was conducted with prior approval from the institutional research ethics committee (registration CE 16/004). Cells were maintained in modified Eagle’s medium (MEM; Sigma-Aldrich, Burlington, MA, USA), enriched with 10% fetal bovine serum (FBS; Sigma-Aldrich), 1% antibiotic solution (1000 IU/mL penicillin and 100 µg/mL streptomycin; Sigma-Aldrich), and 1% L-Glutamine (GlutaMAX; Gibco, Life Technologies, Carlsbad, CA, USA). Cultures were incubated under controlled conditions: 37 ± 1 °C, 95 ± 1% relative humidity and 5 ± 1% CO_2_ atmosphere. For experimental procedures, cells were seeded into 96-well and 24-well plates at an initial density of 1 × 10^5^ cells/mL and incubated for 48 h.

#### 2.2.1. Cytotoxicity Test

The cytotoxic effect of conventional (PMMA) and graphene oxide PMMA (PMMA+GO) was evaluated using hDPSC at a density of 3 × 10^5^ cells/mL. It was tested in two ways: (a) direct contact in 24-well plates, each well containing 0.5 mL of supplemented MEM and a sample from the experimental groups was placed in each well and (b) indirect contact in 96-well plates; for this point an elution was obtained by placing in a Falcon tube 1 mL of MEM supplemented with a sample of each type of conventional PMMA and enriched with graphene oxide, and the samples were placed under sterile conditions in incubation with agitation for 24 h at 180 rpm at 37 °C; after 24 h, the polymer sample was removed and the supernatant (elution) was used as culture medium for the corresponding groups. Finally, the hDPSCs were exposed to direct and indirect contact for 24 h. After that time, the MTT (3-(4,5-dimethylthiazol-2-yl)-2,5-diphenyltetrazole bromide) bioassay was performed using a 0.2 mg/mL solution, and the treated cells were incubated for 4 h with a solution of MTT in fresh culture medium. The formazan formed was dissolved with 0.1 mL dimethyl sulfoxide (DMSO, Karal, Guanajuato, Mexico) for 96-well plates and with 0.3 mL DMSO in 24-well plates, and then an absorbance reading was made on a spectrometer Multiskan GO™ microplate spectrophotometer (Thermo Fisher Scientific, Waltham, MA, USA) at 570 nm. All experiments were performed in triplicate across three independent trials (*n* = 9). Results are presented as the mean percentage of viable cells relative to the control group, which was not exposed to PMMA.

#### 2.2.2. Cell-Proliferation Test

For the cell-proliferation assay, the steps previously described in Section 2.2.1 were performed, but incubation of the samples for both direct and indirect contact was maintained for 3, 7 and 14 days, with fresh supplemented MEM being changed every third day. The experiments were performed in triplicate across three independent trials (*n* = 9). Results are expressed as the mean percentage of viable cells relative to the control group.

#### 2.2.3. Cell-Differentiation Assay

**Figure 2:**
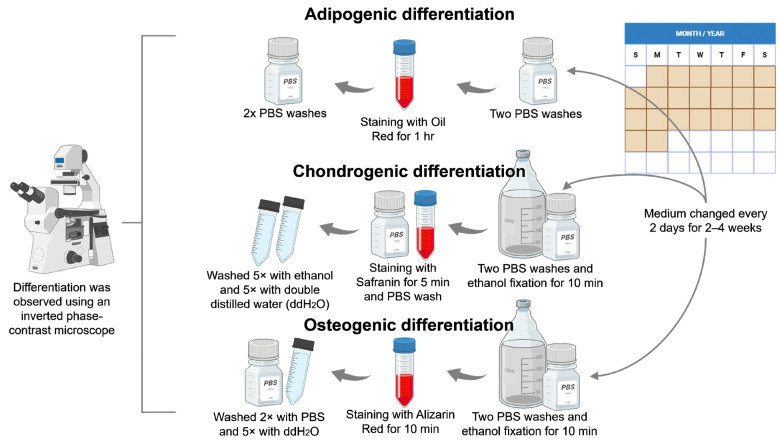
Schematic representation of the method for cell differentiation of hDPSC into adipogenic, chondrogenic, and osteogenic lineage. Source: Direct obtained by BioRender 2025.

Cell subcultures were performed in 24-well plates at a density of 2 × 10^5^ cells/mL. Plates were incubated at 37 °C with 5% CO_2_ and 95% humidity, following the methodology described in Section 2.1.1. for direct and indirect contact with exposure to PMMA and PMMA+GO. Cell-differentiation media were made as follows (Figure 2):

##### Adipogenic Differentiation

After 24 h, the culture medium was removed, and two washes with PBS, 600 µL of adipogenic differentiation medium composed of α-MEM with 10% SFB, 1% penicillin and streptomycin, 0.1 mM dexamethasone, 10 mM B-glycerophosphate, 50 mg/mL ascorbate-2-phosphate, insulin, and L-glutamine were added. Subsequently, the differentiation medium was added and replaced with fresh medium every other day for a period of 4 weeks. At the end of 4 weeks, differentiation was stopped using an oily red stain (0.3 g in 100 mL of 60% *v*/*v* isopropanol) for 1 h at room temperature. Cells were washed twice with PBS; finally, differentiation was observed under a phase contrast microscope.

##### Chondrogenic Differentiation

After 24 h, the culture medium was removed, and two washes with PBS were performed. Subsequently, 600 µL of chondrogenic differentiation medium composed of α-MEM with 10% SFB, 1% penicillin and streptomycin, 0.1 mM dexamethasone, 10 mM B-glycerophosphate, 50 mg/mL ascorbate-2-phosphate, and bone morphogenetic protein (BMP-1 or BMP-4) added. The samples were then incorporated. The differentiation medium was replaced every other day for a period of 2 weeks. At the end of the 2-week period, differentiation was stopped by performing two washes with PBS, and the cells were fixed with 70% *v*/*v* ethanol for 10 min. Subsequently, an additional wash with PBS was performed, and safranin staining (0.15 mL at 0.1%) was applied. Cells were washed five times with distilled water and observed under the microscope.

##### Osteogenic Differentiation

After 24 h, the culture medium was removed, and two washes with PBS were performed. Then, 600 µL of osteogenic differentiation medium composed of α-MEM with 10% SFB, 1% penicillin and streptomycin, 0.1 mM dexamethasone, 10 mM B-glycerophosphate, and 50 mg/mL ascorbate-2-phosphate was added. The PMMA samples were then placed, and the medium was replaced every other day for a period of 4 weeks, until the formation of mineral clusters and birefringent crystals was observed under the microscope. At the end of the 4 weeks, differentiation was stopped by staining with Alizarin red (40 mM in 0.1 M NaH_2_PO_4_ at pH 4.3) for 10 min. Cells were rinsed twice with PBS and fixed with 70% (*v*/*v*) ethanol for 30 min at room temperature. Subsequently, two additional washes were performed with PBS, and 250 µL Alizarin red was added for 10 min. Then, the cells were washed twice with PBS and five times with double distilled water, and finally the cells were observed under a microscope.

### 2.3. Statistical Analysis

Results are reported with the mean and standard deviation (SD) to calculate the cell viability of hDPSCs. Shapiro–Wilks normality tests were performed. Comparative statistics were analyzed according to the distribution of data in the biological tests: cytotoxicity and cell proliferation. For parametric results (ANOVA and Tukey’s post hoc), data were recorded and analyzed with SPSS version 20 software (IBM, Chicago, IL, USA), considering a significance level of *p* < 0.05.

## 3. Results

### 3.1. PMMA+GO Characterization

**Figure 3:**
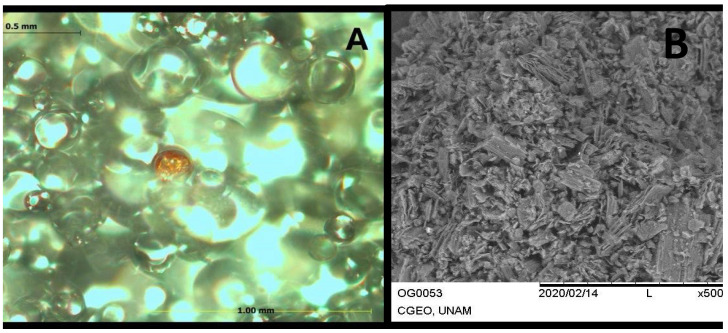
PMMA+GO samples analyzed under an optical microscope, showing spherical particles (**A**); in the SEM photomicrograph, the lamellar morphology of GO is observed (**B**).

As shown by the results previously described by our group [15], in Figure 3A, predominantly spherical particles are observed in PMMA+GO, according to the scale (0.5 mm and 100 µm), their size varying in the range of tens to hundreds of micrometers. The spheres appear to be heterogeneously dispersed, which could indicate a composite sample or different degrees of aggregation. The SEM images show a lamellar or flaky structure, characteristic of GO, and the lamellae are folded, which is common in this type of material. The particle size range is between 15 and 200 µm, and they show a heterogeneous morphology (Figure 3B).

### 3.2. Cytotoxicity Test

**Figure 4:**
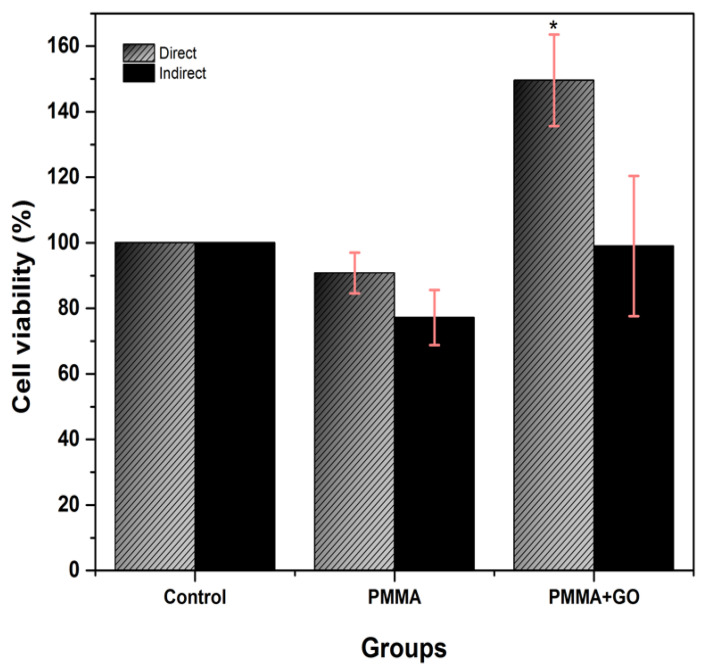
hDPSC cell cytotoxicity in direct and indirect 24 h contact with PMMA and PMMA+GO. Optical density 570 nm. Abs. range (0.137–3.303). Each bar represents the mean percentage ± standard deviation (SD). Statistical analysis was performed using one-way ANOVA, followed by Tukey’s post hoc test. Asterisks (*) indicate concentrations with statistically significant differences compared to the control group (*p* < 0.001, *n* = 9). Source: Direct.

At 24 h of incubation, the PMMA group showed a cell viability of 90.8 ± 6.2% in direct contact and 77.2 ± 8.4 in indirect contact. On the other hand, the PMMA+GO group reached values of 149.6 ± 14% in direct contact and 99.0 ± 21.4% in indirect contact (Figure 4). These results indicate that none of the polymers showed cytotoxicity. Furthermore, it is observed that the addition of GO in the polymer significantly improves cell viability in direct contact (*p* < 0.05), suggesting a possible beneficial effect of GO on cell viability.

### 3.3. Cell-Proliferation Assay

**Figure 5:**
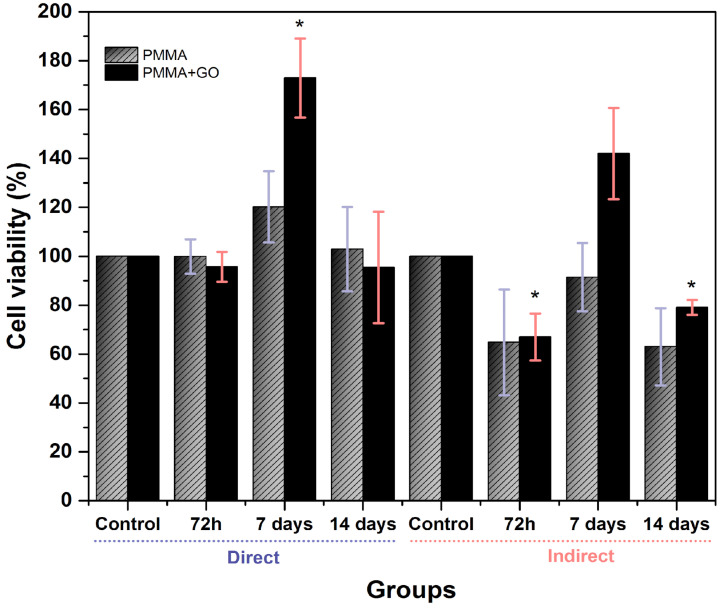
hDPSC cell viability in 72 h, 7 days and 14 days direct and indirect contact with PMMA and PMMA. Optical density 570 nm. Abs. range (0.099–3.214). Each bar represents the mean percentage ± standard deviation (SD). Statistical analysis was performed using one-way ANOVA, followed by Tukey’s post hoc test. Asterisks (*) indicate concentrations with statistically significant differences compared to the control group (*p* < 0.001, *n* = 9). Source: Direct.

In the PMMA group, cell viability in direct contact was 99.9 ± 7.0% at 72 h, 120.2 ± 14.6% at 7 days and 102.9 ± 17.3% at 14 days. In indirect contact, values of 64.8 ± 21.6% at 72 h, 91.4 ± 16.5% at 7 days and 63 ± 15.8% at 14 days were recorded. On the other hand, the PMMA+GO group showed a cell viability of 95.7 ± 6.1% at 72 h, 172.9 ± 16.2% at 7 days and 95.4 ± 22.8% at 14 days in direct contact; while in indirect contact, values of 67 ± 9.6% at 72 h, 142 ± 18.7% at 7 days and 79.1 ± 3.1% at 14 days were obtained (Figure 5). It is worth noting that the presence of GO favors cell viability, particularly at 7 days of incubation, also highlighting the decrease in cell viability (mild cytotoxicity) at 72 h, especially in indirect contact for both polymers (*p* < 0.05).

### 3.4. Cell Differentiation

#### 3.4.1. Adipogenic Differentiation

**Figure 6:**
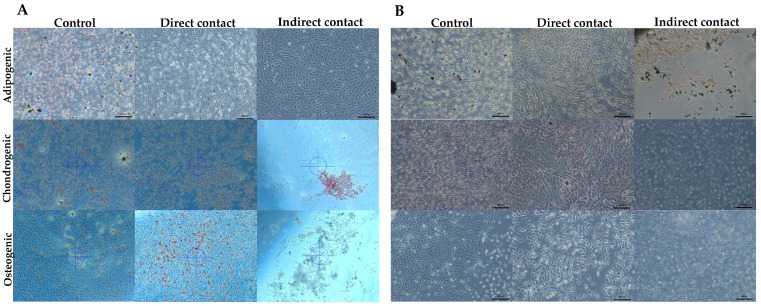
Comparative photomicrographs of hDPSC differentiation into adipogenic, chondrogenic, and osteogenic lineages under control conditions, direct contact, and indirect contact with conventional PMMA (**A**) and GO-enriched PMMA (**B**). Morphological evidence of lineage-specific differentiation is visible in each condition. Source: Direct observation.

The presence of adipocytes, being a qualitative variable, was determined by staining for lipid deposits; 18 samples were observed for each independent assay, to which oil red was applied to show reddish lipid deposits. A greater accumulation of lipid deposits was observed in the samples that were in direct contact with the PMMA added with GO, compared to the samples that were in indirect contact and the samples that did not have GO (Figure 6A,B).

#### 3.4.2. Chondrogenic Differentiation

During the process of chondrogenic differentiation, the matrix secreted by these cells was stained, allowing the detection of glycosaminoglycans and proteoglycans by means of safranin staining. In this differentiation, an increase in chondrogenic differentiation was observed in the group that was in direct contact with the PMMA added with graphene oxide, in comparison with the samples that remained in indirect contact and the samples that did not have GO (Figure 6A,B).

#### 3.4.3. Osteogenic Differentiation

Osteogenic differentiation was assessed by detection of the enzyme alkaline phosphatase, a marker of the osteoblastic phenotype, and extracellular matrix mineralization, which serves as a marker of functionality. A total of 18 samples per independent assay were observed under phase contrast microscopy, and mineral clusters and birefringent crystals were identified under the microscope, with alizarin red staining, in the PMMA+GO samples that remained in indirect contact in addition to the unmodified direct contact samples, whereas the graphene oxide-modified samples belonging to the direct group in addition to the indirect contact samples that had not been modified with GO did not show a significant difference (Figure 6A,B).

**Figure 7:**
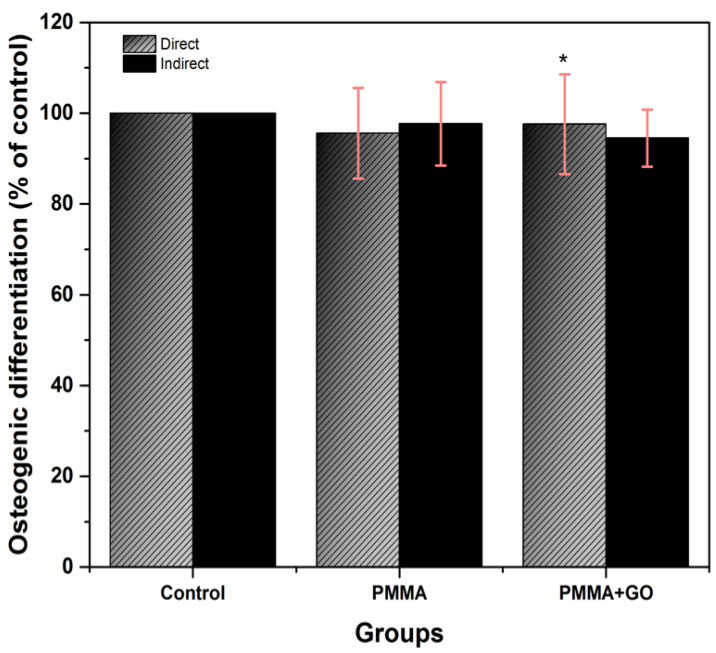
Osteogenic differentiation in direct and indirect contact with PMMA and PMMA. Optical density 570 nm. Abs. range (0.045–0.052), ach value in the graph represents percentage of the mean and SD. One-way ANOVA was performed, Tukey’s post hoc, (*) represents concentrations with significant difference. Data represent mean and variance. ANOVA test * *p* < 0.05, Tukey’s post hoc, *n* = 9. Source: Direct.

The samples of this assay were taken to a microplate reader (Thermo Fisher Scientific, Helsinki, Finland) in which a reading was made at 550 nm, and the corresponding data were analyzed with ANOVA, finding a value of *p* < 0.05, where a statistically significant difference could be observed in the samples that remained in indirect contact with graphene and in direct contact without GO (Figure 7).

## 4. Discussion

The results of this research show a significant increase in cell differentiation in hDPSCs exposed to PMMA enriched with GO. This phenomenon can be attributed to the functional groups present in GO, which allow the formation of covalent and non-covalent bonds with other molecules, creating preconcentration platforms for chemical inducers that promote cell-differentiation processes. These findings are consistent with those reported by Lee et al., 2011, who observed an increase in cell differentiation toward adipogenic and chondrogenic lineages on GO substrates due to their ability to act as a preconcentration platform [23,24,25,26,27].

The results obtained in this research highlight the decrease in cell viability at 72 h, especially in indirect contact of both experimental groups, and this response could be related to an initial phase of cell adaptation to the material environment. It is common that, in the first hours of exposure to new materials, cells experience some stress due to the release of residual compounds or the need to adjust their metabolism and adhesion to the surface. Possibly this decrease is related to an initial release of polymerization by-products or interaction with the medium. However, the recovery observed in the following days, with a significant increase in viability, indicates that this effect is not permanent and that the cells can successfully proliferate over time [23].

In addition, recent studies have confirmed the ability of GO to enhance cell viability and proliferation in materials such as PMMA. Mirza et al., 2019 [28], demonstrated higher viability and a more effective osteogenic response in GO-modified PMMA, while Pahlevanzadeh et al., 2021 [29], concluded that GO-reinforced PMMA-based cement exhibited superior bioactive characteristics compared to conventional PMMA. Similarly, Garcia-Contreras et al., 2021 [14], reported better proliferation of osteoblasts in contact with PMMA+GO, highlighting their potential to stimulate cell proliferation.

In terms of cell differentiation, this research shows complementary results to those of Soleymani et al., 2020, who showed a faster recovery in tissues thanks to the bioactivity of GO in complex bone cavities, and to those of a more recent study by Krukiewicz et al., 2020, who reported an increase in the osteogenic markers ALP, SPARC, and BMP-2 when using PMMA modified with GO [30,31]. However, the results of this work indicate that, although a significant increase in osteogenic differentiation was not observed, there was an increase in adipogenic and chondrogenic differentiation in the group treated with PMMA enriched with GO. This behavior can be explained by the specific interactions between the –OH groups of GO and molecules such as ascorbic acid, through hydrogen bonds that facilitate the attraction and binding of chemical inducers. These results are encouraging for the design of biomaterials in tissue engineering, as they confirm the role of GO as a key component in improving the bioactive properties of PMMA-based materials, especially for regenerative applications where specific cell differentiation is desirable. Despite the non-uniform spherical morphology observed in PMMA+GO, no adverse effects on hDPSC viability or differentiation were detected. On the contrary, the results showed an increased metabolic activity and enhanced proliferation, particularly in direct contact conditions. These findings suggest that the surface chemistry and oxygenated functional groups of GO may facilitate cellular adhesion and interaction, outweighing any negative influence from the irregular morphology. Furthermore, although both PMMA and PMMA+GO fulfilled the ISO 10993-5 [32] cytotoxicity threshold of ≥75% cell viability, the consistently higher values observed in PMMA+GO—especially at 7 days—point to a potential bioactive role of GO in promoting a more favorable microenvironment for cell survival and growth. These results align with previous studies reporting improved osteogenic, chondrogenic, and adipogenic responses when GO is incorporated into polymeric matrices [14,30].

## 5. Conclusions

The findings of the present investigation underline the promising potential of incorporating GO into PMMA for applications in regenerative medicine and dental materials. The modification of PMMA with graphene oxide not only improves its bioactive properties, such as the differentiation of hDPSC toward osteogenic, adipogenic, and chondrogenic lineages, but also favors cell proliferation, evidenced by the MTT assay. These characteristics position it as an optimal material for tissue regeneration and the design of biomedical devices.

Regarding safety, it is highlighted that PMMA enriched with graphene oxide does not present significant cytotoxicity compared to conventional PMMA, since it maintains a cell viability percentage ≥75% in direct and indirect contact evaluations with hDPSC. This result reinforces the possibility of its safe clinical application.

While the in vitro results are encouraging, future in vivo studies will be crucial to validate the behavior of this material in complex biological environments. If its efficacy and safety are confirmed, graphene oxide-modified PMMA could constitute a valuable tool in the regeneration of maxillofacial defects, the manufacture of prostheses, and even in other applications of tissue engineering and regenerative medicine. Thus, this innovation could represent a significant advance in the development of multifunctional biomedical materials.

## Data Availability

The raw data supporting the conclusions of this article will be made available by the authors on request.
